# The *PHOX2B* c.428A>G missense variant affects post-transcriptional regulation and may explain the absence of neural crest-derived tumors in congenital central hypoventilation syndrome

**DOI:** 10.3389/fphys.2025.1616994

**Published:** 2025-09-22

**Authors:** Tiziana Bachetti, Simona Bagnasco, Giuseppe Santamaria, Maria Francesca Bedeschi, Francesca Menni, Igor Catalano, Luigina Spaccini, Francesco Cavigioli, Francesco Morandi, Isabella Ceccherini

**Affiliations:** ^1^ IRCCS Ospedale Policlinico San Martino, Genoa, Italy; ^2^ IRCCS Istituto Giannina Gaslini, Genoa, Italy; ^3^ Fondazione IRCCS Ca’ Granda Ospedale Maggiore Policlinico, Milan, Italy; ^4^ Pediatric Palliative Care Service, VIDAS ODV, Milan, Italy; ^5^ Ospedale dei Bambini Vittore Buzzi, Milan, Italy; ^6^ Italian Association for Congenital Central Hypoventilation Syndrome (A.I.S.I.C.C.), Firenze, Italy

**Keywords:** paired-like homeobox 2b, transcript splicing, Hirschsprung disease, neural crest-derived tumors, loss-of-function

## Abstract

**Introduction:**

Heterozygous mutations in the paired-like homeobox 2b (PHOX2B) gene cause congenital central hypoventilation syndrome (CCHS). While polyalanine expansions are almost exclusively associated with isolated CCHS, missense, nonsense, and frameshift mutations are mainly identified in syndromic CCHS, presenting with Hirschsprung disease (CCHS + HSCR) alone and/or together with neuroblastoma (CCHS + HSCR + NB). CCHS-associated missense mutations occur in the PHOX2B homeodomain, where impaired transcriptional activity has been suggested as their functional effect. However, the molecular pathogenesis underlying their association with HSCR- and/or NB-associated CCHS has not been investigated to date.

**Methods:**

we applied an *in silico* analysis and a minigene approach in vitro to test the effect of the *PHOX2B* c.428A>G missense variant on the splicing of intron 2.

**Results:**

we demonstrate that the missense c.428A>G variant, reported by us and others in a set of CCHS + HSCR cases but never associated with NB, not only causes the amino acid change p.Q143R change but also disrupts the intron 2 splice donor site, producing an aberrant mRNA transcript and likely a hypomorphic, dysfunctional protein.

**Discussion:**

We, therefore, propose that in the presence of splicing defects of PHOX2B, a loss-of-function mechanism may underlie CCHS + HSCR and potentially explain the absence of neural-crest-derived tumors.

## 1 Introduction

Heterozygous mutations in *PHOX2B*, a gene encoding a transcription factor expressed during the differentiation of neural crest cells (NCCs) into autonomic nervous system precursor cells (Pattyn et al., 1999), are associated with congenital central hypoventilation syndrome (CCHS). CCHS patients are characterized by an abnormal ventilatory response to hypoxia and hypercapnia, requiring treatment with ventilatory support, tracheostomy, or diaphragmatic pacers, especially during sleep.

The vast majority of mutations consist of expansions, ranging from +4 to +13 residues, of a 20-residue polyalanine stretch in exon 3, whose pathogenic effect is expressed through the partial or total retention of PHOX2B in the cytoplasmic compartment, in a soluble or aggregated form, thus compromising its role in the transcriptional regulation of target genes exerted in the nucleus (Bachetti et al., 2005a; Bachetti and Ceccherini, 2020). However, modulation of the cellular response using Hsp90 inhibitors and histone deacetylase (HDAC) inhibitors has shown beneficial effects *in vitro* (Di Zanni et al., 2012; Africano et al., 2024).

Life-threatening breathing deficiencies in CCHS can be accompanied by Hirschsprung disease (HSCR)—a condition marked by intestinal aganglionosis due to defective migration of NCC precursors of enteric ganglion cells—NCC-derived tumors such as neuroblastoma (NB) (Trochet et al., 2005), and several other autonomic nervous system dysfunctions (Trang et al., 2020). CCHS can, therefore, manifest as isolated or syndromic, with the latter most often characterized by the co-occurrence of HSCR with or without NB. Interestingly, *PHOX2B* mutations are uniquely responsible for syndromic forms of CCHS, in contrast to atypical/isolated presentations, which may be associated with different genetic alterations (Spielmann et al., 2017; Hernandez-Miranda et al., 2018).

While polyalanine repeat mutations (PARMs) are mainly associated with isolated CCHS, syndromic CCHS—particularly CCHS + HSCR or CCHS + HSCR + NB—is more frequently associated with non-PARMs (NPARMs), including missense (MS), nonsense (NS), and frameshift (FS) mutations (Bachetti and Ceccherini, 2020).

Frameshift mutations are differently distributed along the *PHOX2B* gene in patients with CCHS + HSCR, with or without NB, depending on the type of translational frame disruption. This disruption affects the severity of the resulting transcriptional dysfunction and the predisposition to isolated or syndromic CCHS (Di Lascio et al., 2018). Moreover, both loss-of-function and gain-of-function effects, with the latter characterized by aberrant target gene acquisition such as *SOX10* and *GFAP* (Nagashimada et al., 2012; Di Lascio et al., 2018) and the failure of sympathoadrenal progenitor cells to differentiate into the neuronal lineage (Ponzoni et al., 2022), have been proposed for NB occurrence in the presence of FS mutations.

Conversely, most missense mutations affect the homeodomain region, leading to different syndromic CCHS associations, whose underlying molecular mechanisms are still undisclosed.

Starting from the molecular and clinical characterization of two patients carrying the *PHOX2B* c.428A>G (p.Q143R) variant, affected by CCHS and intestinal abnormal phenotypes, in this work, we have investigated how the nucleotide change and substitutions at neighbor positions, rather than the amino acid replacement itself, could influence the clinical expressivity of homeodomain-affecting *PHOX2B* variants.

## 2 Methods

### 2.1 CCHS molecular diagnosis

The molecular analysis of the *PHOX2B* gene was performed at IRCCS Giannina Gaslini.

For patient 1 (MN), the coding region of the *PHOX2B* gene was analyzed for mutations by direct DNA sequencing using the BigDye Terminator Cycle Sequencing Kit (Applied Biosystems) on an ABI 3100 DNA Automated Sequencer (Matera et al., 2004), according to a protocol we have already reported.

For patient 2 (SM), whole-exome sequencing was performed, as already reported (Di Rocco et al., 2018), using the Ion Torrent technology with coverage >100X (Ion-Proton, Thermo Fisher Scientific) on DNA extracted from peripheral blood. The *PHOX2B* variant thus detected was confirmed by direct DNA sequencing, as described above.

In both patients, the c.428A>G variant in exon 2 of the *PHOX2B* gene was detected in heterozygosity.

### 2.2 *In silico* splicing prediction software

*In silico* prediction of splicing at the *PHOX2B* gene was performed using both the Alternative Splice Site Predictor (ASSP) (http://wangcomputing.com/assp/) and the SD-score algorithm for mutation simulation (https://www.med.nagoya-u.ac.jp/neurogenetics/SD_Score/sd_score.htmli). The ASSP is a sequence analysis tool for the prediction and classification of splice sites. The ASSP identifies putative splice sites using pre-processing models and subsequently classifies them as either constitutive or alternative isoform/cryptic splice sites, combining several statistics, such as position-specific score matrices for the splice sites, GC content, and oligonucleotide frequency models (Wang and Marin, 2006). The classification performance of ASSP, i.e., the distinction between constitutive or alternative isoform/cryptic splice sites, is 67.45% for the acceptor sites and 71.23% for the donor sites. The SD-score algorithm is a practical tool to study the splicing consequences of mutations affecting the 5′splice site *in silico*, predicting aberrant splicing when the SD score for the mutant allele is lower than that for the wild-type (WT) allele (Sahashi et al., 2007). The SD score is accompanied by the information content (Ri), which improves predictive accuracy. Differences between two sequences are expressed as “delta,” with ΔSD < −0.34 and ΔRi < −1.45 indicating aberrant splicing (Sahashi et al., 2007).

### 2.3 Generation of WT (c.428A) and mutant (c.428G) pSPL3 constructs

After evaluating the *in silico* splicing prediction for the c.428A/G alleles, a 261 bp region in the *PHOX2B* gene—encompassing exon 2 and 45 bp upstream and 28 bp downstream of flanking intronic sequences—was amplified from the gDNA of a patient heterozygous for the c.428A>G variant using the AccuPrime GC-Rich Kit (Life Technologies) and primers Ph2F 5′-TGCCGGCTGATTTGCTCAC-3′ and Ph2R 5′-AGCGGGGTCGGTTTCCAGG-3’. PCR products were then cloned into the pCR2.1 vector (TOPO TA cloning, Life Technologies) to separate the A and G alleles. The A and G fragments were EcoRI-digested from TOPO TA plasmids to be inserted into the EcoRI site of the pSPL3 plasmid to generate the two pSPL3-A and pSPL3-G minigenes.

### 2.4 Cell cultures and transfection

Although not of neural crest origin, HEK293T cells were selected to test the effect of the “G” allele on *PHOX2B* gene splicing because i. they are characterized by high transfection efficiency, which is necessary for performing functional studies using transient transfections, and ii. splicing is a ubiquitous mechanism, which does not require a specific cellular background. This cell line was grown in DMEM supplemented with 10% FBS, 1% glutamine, and 1% penicillin/streptomycin. Cells were plated at 70% confluency the day before transfection, which was performed by mixing 5 μg of DNA from pSPL3-A and pSPL3-G constructs with FuGENE HD (Promega) at a 3:1 ratio.

### 2.5 Splicing minigene assay

Five micrograms of pSPL3-A and pSPL3-G plasmids were transiently transfected into HEK293T cells, and total RNA was extracted 24 h later (RNeasy Plus mini Kit, QIAGEN). cDNA was obtained starting from 1 μg RNA (iScript cDNA Synthesis Kit, Bio-Rad). Polymerase chain reaction (PCR) on cDNA was performed using primers SD6 (5′-TCT GAG TCA CCT GGA CAA CC-3′) and SA2 5’-(ATC TCA GTG GTA TTT GTG AGC-3′) and AccuPrime GC-Rich DNA Polymerase Kit (Thermo Fisher Scientific) in 25 μL reaction mixture through 40 cycles at 95 °C for 10′, 95 °C for 30″, 55 °C for 45″, and 72 °C for 3′, followed by one cycle at 72 °C for 7’. The PCR products were then analyzed via electrophoresis on a 1.5% agarose gel and characterized by Sanger sequencing using the SD and SA2 primers.

### 2.6 Quantification and statistical analysis

The percentage of correctly or aberrantly spliced transcripts was estimated by quantifying the bands from the electrophoresis analysis of PCR products using ImageJ, particularly using the ratio between the intensity of the target band and the combined intensity of the upper and lower bands of the gel. The results were analyzed using GraphPad Prism. Comparisons of the means between the two groups were performed using two-way ANOVA, followed by Sidak’s multiple testing correction.

## 3 Results

### 3.1 Clinical evaluation of CCHS patients carrying the *PHOX2B* c.428A>G variant

*Patient 1*. MN, a female child born prematurely at 34+1 weeks via cesarean section, presented with central hypoventilation and hypotonia at birth associated with pulmonary hypertension. She was immediately transferred to the Neonatal Intensive Care Unit at Buzzi Hospital due to severe respiratory acidosis and was treated with intubation, mechanical ventilation, and, after a month of hospitalization, tracheostomy. At the age of 1 month, the identification of the c.428A>G variant in the *PHOX2B* gene, leading to the amino acid change p.Q143R, confirmed the molecular diagnosis of CCHS. The mutation occurred *de novo*, being absent in both parents. Moreover, preliminary observations of severe constipation and abdominal distension suggested rectal biopsies that confirmed the lack of ganglionic structures in the submucosa, thus leading to the diagnosis of co-occurring HSCR. Currently, after several hospitalizations for the removal of the intestinal aganglionic tract, hypoglycemia, and intestinal perforation due to necrotizing enterocolitis (NEC), she is under continuous respiratory, nutritional, and neurological monitoring. Finally, she suffered from essential arterial hypertension, which was treated with captopril, and severe hypermetropia.

*Patient 2*. SM, a baby girl born at 36 weeks by cesarean section for alterations in cardiotocographic tracing, was immediately transferred to the Neonatal Intensive Care Unit of the Rho Hospital (Milan) for the absence of spontaneous respiratory activity and hypotonia and subsequently to the Neonatal Intensive Care Unit, Fondazione IRCCS Ca’ Granda Ospedale Maggiore Policlinico (Milan). She presented with early feeding difficulties and delayed neurological development, with episodes of withdrawal of contact, sialorrhea, generalized hypertonicity, and limb clonus. The EEG during sleep showed a “slow trend compatible with epileptiform episodes,” which subsequently normalized without requiring continuous anti-epileptic therapy. The child was then taken to the pediatrics department of the De Marchi Clinic in Milan, and at 4 months, she was treated with a tracheostomy to allow mechanical ventilation during sleep. During the first year of life, the child also experienced recurrent hyperinsulinemic hypoglycemic episodes, as confirmed by a pathological glucagon test. In the subsequent years, hyperglycemia occurred during intercurrent infections. Concomitant to the metabolic imbalance, the child presented signs of intestinal dysmotility with alternate alvo. Detection of the c.428A>G variant in the *PHOX2B* gene confirmed that the child had CCHS. Again, the mutation had occurred *de novo*, being absent in both parents. After a few years, she was admitted to the Buzzi Hospital in Milan for signs of intestinal dysmotility, such as marked constipation with some sub-occlusive episodes. She presented with abdominal pain and a distended abdomen in the upper quadrants. Furthermore, her body growth was delayed. Two years later, she was evaluated for gastrointestinal disorders due to severe episodes of diarrhea; following two aspiration biopsies, the quality of which did not allow excluding or confirming the presence of ganglia, focal hypertrophy of cholinergic fibers in the muscularis mucosae and lamina propria was observed, ultimately suggesting ultra-short HSCR disease. The metabolic changes gradually resolved as intestinal motility and growth improved.

### 3.2 *In silico* prediction of *PHOX2B* splicing defects due to c.428A>G

**FIGURE 1:**
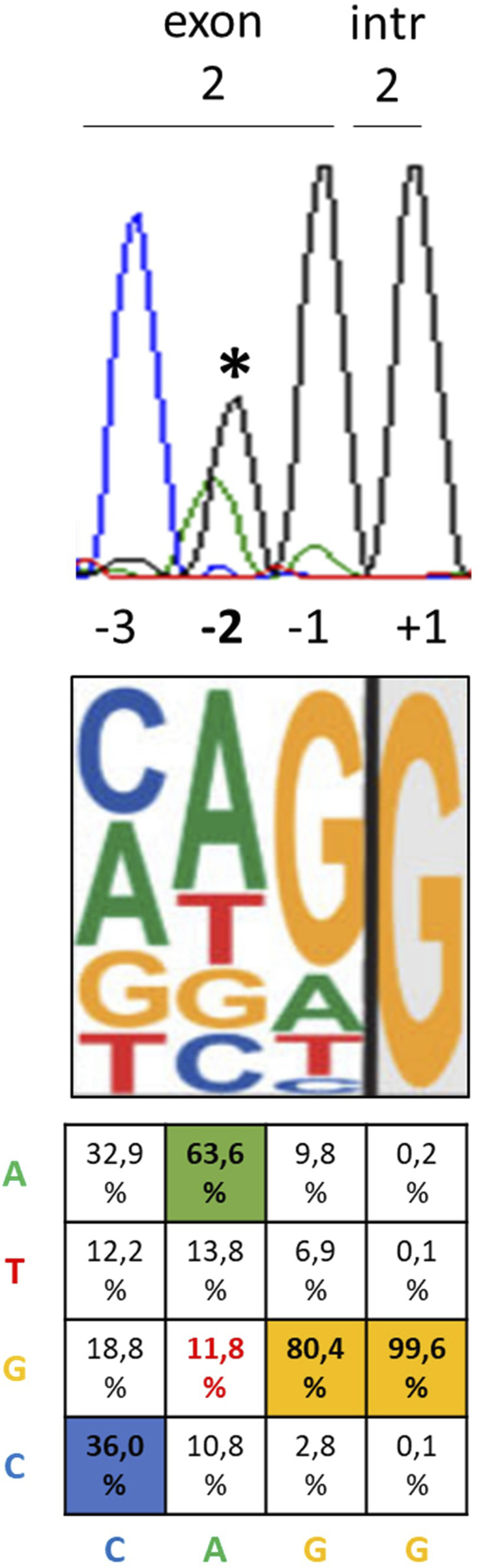
Position of the c.428A>G *PHOX2B* variant relative to the exon–intron junction and most likely nucleotides. From top to bottom, the three pictures represent the electropherogram showing the heterozygous c.428A>G variant in the DNA sequence of the CCHS + HSCR patient (asterisk) with the position of each nucleotide with respect to the exon 2–intron 2 boundary, the most probable consensus matrix of the 5′ splicing site (modified by Sibley et al., 2016), and the probability (%) to find A, T, G, and C nucleotides in each position from −3 to +1 (Sahashi et al., 2007).

After observing that the c.428 nucleotide represents the second-last position of exon 2, namely, -2 nt with respect to the GT donor site of intron 2 (Figure 1), we hypothesized a role for the c.428A>G variant in the regulation of the splicing process. At the -2 nt position, A is present in 63.3% of cases, while G has been found only in 11.8% of cases (Sahashi et al., 2007; Sibley et al., 2016) (Figure 1).

To investigate whether the c.428G allele could interfere with the splicing process, the entire *PHOX2B* exon 2, flanked by portions of introns 1 and 2, underwent *in silico* analyses using the ASSP and SD-score algorithms. Both programs suggested aberrant splicing in the presence of the c.428G allele compared to the A allele. In particular, the ASSP detected a lower splicing score for the G allele than that for the A allele, in addition to an increased value of activation of a cryptic splicing site (Table 1). The SD score showed ΔSD = −1,009 and ΔRi = −2,456, which are largely below the threshold of the values predicting aberrant splicing, namely, ΔSD < −0.34 and ΔRi < −1.45 (Table 1). In addition, ESEfinder 2.0 further confirmed the results obtained using the two other software applications (not shown).

**TABLE 1:** *In silico* analysis, through the ASSP and SD-score algorithms, of the possible effect on splicing of the c.428A>G PHOX2B variant.

SD-score	Sequence	Score^a^	ΔSD score	ΔR_i_	Prediction
	cAggtacgc	−3.092	−1.009	−2.456	Aberrant
	cGggtacgc	−4.101			

ASSP	sequence	Score^a^	Alt./Cryptic	Constitutive	Confidence
	GCGAGTCCAGgtacgcgcgc	10.028	0.652	0.267	0.590
	GCGAGTCCGGgtacgcgcgc	6.281	0.838	0.120	0.856

^a^ Scores of the models reflect splice site strength.

For the SD score: ΔSD < −0.34: aberrant splicing; ΔRi < −1.45: aberrant splicing.

Constitutive: exons included in the normal splice form.

Cryptic exons: only exceptionally included exons.

Alt.: exon isoforms derived from truncated or extended exon isoforms resulting from the use of an alternative splice site.

ASSP donor threshold: 0.71.

### 3.3 Functional effect of c.428A>G on the splicing process

**FIGURE 2:**
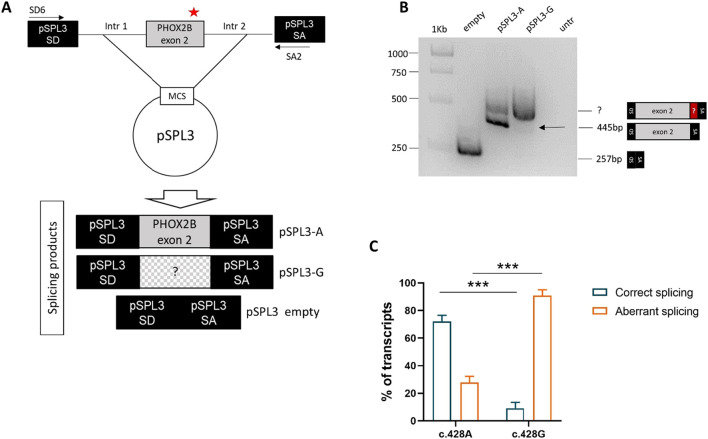
Evaluation of the splicing effect of the c.428A>G variant by gel electrophoresis following the pSPL3 minigene assay. **(A)** Cloning strategy of the genomic region in pSPL3 (the position of the c.428A>G variant is indicated by a red star). **(B)** Agarose gel shows the SD6-SA2 PCR products derived from HEK293T cells transfected with the empty pSPL3 vector (lane 1), the pSPL3-A (c.428A allele, lane 2) and pSPL3-G (c.428G allele, lane 3) constructs, in addition to untransfected cells (lane 4). The size of the PCR products is indicated on the right. Correct splicing of exon 2 generates a 445 bp fragment. **(C)** Quantification of the bands corresponding to the aberrant and correct splice products for each of the two alleles (A and G) is shown below.

The *PHOX2B* exon 2, flanked by its intronic regions, was cloned into the pSPL3 expression vector between the two artificial splice donor (SD) and splice acceptor (SA) sites, thus making the minigenes *pSPL3-A* and *pSPL3-G* suitable for testing the splicing efficiency of the subcloned region. In particular, we transfected the human HEK293T cell line, characterized by high transfection efficiency, with the constructs bearing the wild-type *PHOX2B* allele c.428A or the variant c.428G, in addition to the empty pSPL3 construct, containing only the SD- and SA-flanked endogenous exon and used as the positive control to assess the efficiency of the *in vitro* splicing process. Following RNA extraction and cDNA synthesis, PCR amplification of the transcripts was performed using primers that map to the vector (Figure 2A).

**FIGURE 3:**
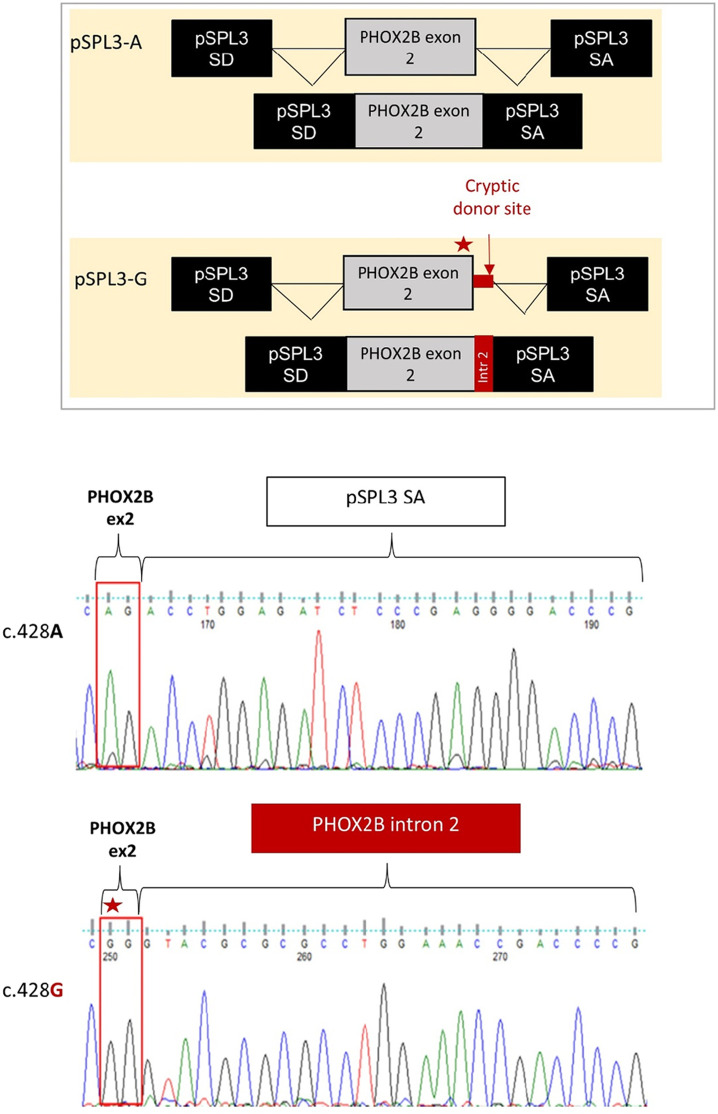
Evaluation of the splicing effect of the c.428A>G variant by Sanger sequencing following the pSPL3 minigene assay. From top to bottom it is schematized i) the correct joining between *PHOX2B* exon 2 and the downstream pSPL3 exon SA for the c.428A allele (pSPL3-A); ii) the aberrant splicing between *PHOX2B* exon 2 and the downstream pSPL3 intron, generated by the abolition of the canonical donor site with the subsequent activation of a criptic GT donor site within the intron; iii) the electropherogram of the splice fragment sequences showing the splicing products induced by both the c.428A and c.428G alleles in the pSPL3 minigene assay.

Gel electrophoresis analysis of PCR products showed that, while no transcript could be detected by the cDNA analysis of the HEK293T not-transfected cells, the empty pSPL3 was able to correctly perform the splicing of the sequence lying between SD and SA, as demonstrated by the 257 bp product derived from the SD–SA joining fragment (Figure 2B). Moreover, while the presence of the wild-type c.428A allele allowed mostly correct splicing, generating the expected 445 bp transcript, the c.428G allele produced a larger transcript fragment in the absence of the wild-type fragment, thus suggesting the occurrence of an aberrant splicing. Quantification of gel electrophoresis bands of three different biological replicates confirmed that almost 80% of the pSPL3-A vector underwent correct splicing, with only 20% of the transcripts using a cryptic donor site; in contrast, in the presence of the pSPL3-G plasmid, more than 90% of the transcripts were generated using the cryptic donor site, and in less than 10% of the cases, the splicing process occurred correctly (Figure 2C). The sequencing analysis eventually confirmed that the presence of the c.428G allele caused the inactivation of the functional intron 2 splice donor site and the aberrant inclusion of part of the PHOX2B intron 2 into the final transcript (Figure 3).

## 4 Discussion

In this work, we report for the first time the functional consequence of a *PHOX2B* c.428A>G (p.Q143R) variant, an NPARM found in two patients from our cohort, associated with syndromic CCHS co-occurring with intestinal dysfunctions. In particular, we show that the missense variant masked a splicing defect and its consequences.

In contrast to the vast majority of missense mutations affecting exon 2, which are associated with neural crest cell tumors (NCTs), such as neuroblastoma, either isolated or occurring with CCHS, the CCHS patients in this study showed no evidence of tumors. Furthermore, several CCHS patients carrying the same missense substitution c.428A>G with co-occurrence of only HSCR have already been reported (Table 2) (Trochet et al., 2005; Berry-Kravis et al., 2006; Nabila et al., 2016; Kasi et al., 2021; Balakrishnan et al., 2021), thus confirming that no NCT associated with this *PHOX2B* mutation has ever been found, with the exception of one benign ganglioneuroma that had to be resected (Balakrishnan et al., 2021).

**TABLE 2:** Patients already reported to carry the c.428A>G *PHOX2B* variant with associated symptoms and references.

Reference	Patient	PHOX2B gene variant (NM_003924.3)	Clinical presentation	Age at diagnosis	Parental PHOX2B origin	Hirschsprung disease (HSCR)	Neural crest tumor
Trochet et al. (2005)	O.82	c.428A>G (p.Gln143Arg)	Hypoventilation			+ (L-HSCR)	-
Berry-Kravis et al. (2006)	3	c.428A>G (p.Gln143Arg)	Hypoventilation			+	-
Berry-Kravis et al. (2006)	4	c.428A>G (p.Gln143Arg)	Hypoventilation			-	-
Nabila et al. (2016)	case report	c.428A>G (p.Gln143Arg)	Severe hypoventilation			-	-
Kasi et al. (2021)	4	c.428A>G (p.Gln143Arg)	Apnea and cyanosis at birth	5 weeks	*De novo*	+	Adrenal ganglioneuroma (resected)
Balakrishnan et al. (2021)	-	c.428A>G (p.Gln143Arg)	Hypoventilation			+ (L-HSCR)	-
this paper	1	c.428A>G (p.Gln143Arg)	Severe hypoventilation	At birth	*De novo*	+	-
this paper	2	c.428A>G (p.Gln143Arg)	Absence of spontaneous respiration, feeding difficulties, and delayed neurological development	At birth	*De novo*	Constipation with sub-occlusive episodes	-

L-HSCR, long-segment Hirschsprung disease.

The hypothesis of an involvement of the c.428A>G variant in the splicing process was postulated when NPARMs were first associated with syndromic CCHS (Berry-Kravis et al., 2006), but it has never been demonstrated to date. Our results show that the c.428G allele, due to its proximity (−2) to the end of exon 2, can alter the splicing process, thus impairing the production of correct mutant mRNA. However, in contrast to what was postulated by Berry-Kravis et al. (2006), we suggest that the aberrant mRNA product may produce a hypomorphic protein, likely leading to a loss-of-function rather than a toxic gain-of-function effect. Unfortunately, given the unavailability of CCHS target tissues, we were unable to verify whether the same aberrant splicing also occurs in patient cells. Furthermore, we were unable to characterize the resulting abnormal protein *in vitro* or assess whether mRNA surveillance mechanisms could downregulate or suppress mutant mRNA levels.

Given the well-known role of *RET* haploinsufficiency in HSCR pathogenesis, caused by *RET* mutations with loss-of-function or downregulation of *RET* gene expression (Edery et al., 1994), and the role of PHOX2B in promoting and regulating *RET* expression, the latter can probably be compromised in the presence of *PHOX2B* mutations that are no longer able to transactivate the *RET* promoter (Pattyn et al., 1999; Bachetti et al., 2005b; Di Zanni et al., 2017), namely, in the presence of a hypomorphic PHOX2B protein.

Therefore, one could hypothesize that a loss-of-function mechanism due to reduced function/expression, rather than a gain-of-function mechanism, underlies the *PHOX2B* NPARMs effects in the absence of NCC-derived tumors. This is in agreement with a recent report of a three-generational familial case in which a mutation in intron 1, identified in three patients with CCHS + HSCR, was predicted to alter the splicing process (Pace et al., 2020). Although authors claim the possibility that, similarly to nonsense mutations in exon 1, a cryptic translational start codon is used (Cain et al., 2017), they suggest that production of a hypomorphic protein, rather than haploinsufficiency due to nonsense-mediated decay, may occur. However, since they do not analyze the consequence of such a splicing variant in intron 1 with a minigene, they cannot exclude that nonsense-mediated decay may partially eliminate the mutant gene product.

It is interesting to note that the variant c.422G>A (p.R141Q) is located within the sequence coding for the homeodomain at position (−8) with respect to the last nucleotide of exon 2; therefore, it is not close enough to be predicted by the software analysis to interfere with splicing, and it has been found in several patients suffering from syndromic CCHS, including neuroblastoma (Trochet et al., 2005; Lombardo et al., 2018). This observation seems to support the hypothesis of different pathogenetic mechanisms underlying NB occurrence in syndromic CCHS.

**FIGURE 4:**
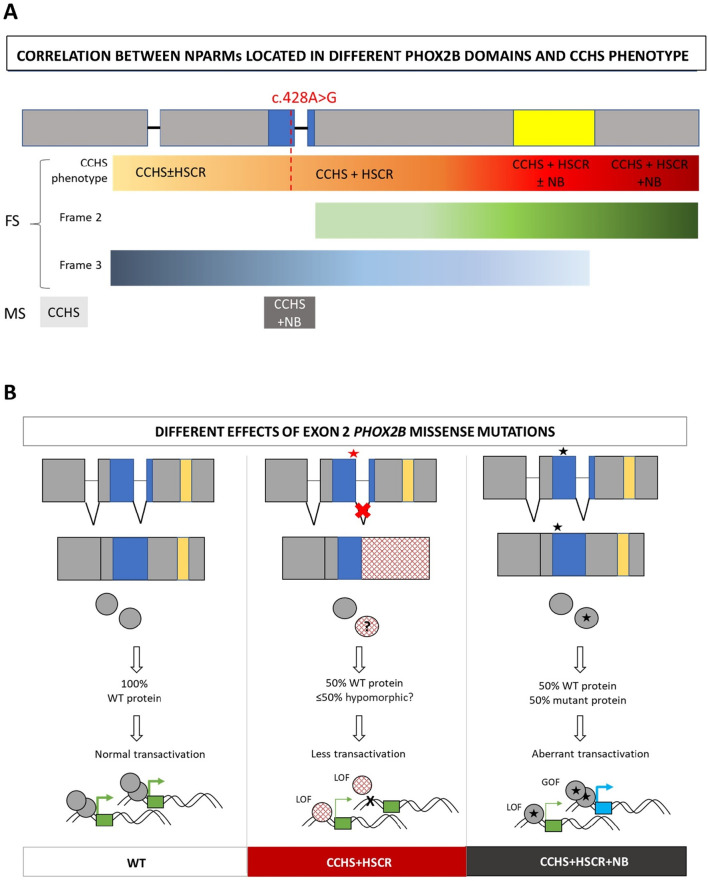
Hypothesis on the mechanism of action of splicing mutations in syndromic CCHS. **(A)** Distribution along the gene domains of frameshift (FS) and missense (MS) *PHOX2B* variants in association with the different phenotypes. The symbol ± indicates whether or not you are likely to have the phenotype. The intensity of the color from yellow to dark red indicate the increasing severity of CCHS phenotypes, starting from isolated CCHS to syndromic with different degrees of severity. The red line correspond to the location of the c.428A>G mutation and its corresponding phenotype in the hypothesis of a frameshift effect of the splicing defect. Moreover, the intensity of the green (frame 2) and blue (frame 3) bars correlates with the distribution of FS with frame 2 and frame 3, respectively. For missense (MS), isolated CCHS and CCHS + NB are indicated in light and dark grey, respectively. **(B)** From left to right: correct splicing of exon 2, aberrant splicing of exon 2 mediated by MS variants in CCHS+HSCR, correct splicing of exon 2 in the presence of MS variants inducing CCHS + HSCR + NB. The molecular consequences of the three cases are shown. Red star: splicing defect induced by MS mutation; Black star: no splicing defect induced by MS mutation. Blue box: homeodomain; yellow box: polyalanine region. LOF: loss-of-function; GOF: gain-of-function. Squared circles (mid panel): disrupted mutant protein due to aberrant splicing, likely reduced with respect to the WT due to quality control systems (nonsense mediated decay for mRNA or ribosome quality control for elongated mutant peptides); star circles (right panel): full length mutant protein carrying the MS mutation. The possible effects of the WT and mutant products on target gene transactivation are also shown. Green boxes: target genes regularly transactivated by the WT; blue box: aberrantly acquired target gene; thick arrows: active promoter transactivation; thin arrows: lower promoter transactivation.

The position of the c.428A>G variant and the results presented in this study, suggesting an FS mutation masked by MS, are in accordance with the reported “regional” distribution of FS mutations underlying CCHS + HSCR or CCHS + HSCR + NCC tumors. We have already observed that CCHS + HSCR alone mostly associates with changes in the reading frame arising upstream of the polyAla region, while the triple phenotype is predominantly associated with variants affecting the polyAla region (CCHS + HSCR ± NB) and is almost exclusively associated with variants located downstream of the polyAla tract (CCHS + HSCR + NB) (Di Lascio et al., 2018), with an NB-promoting gradient in this distal region (Figure 4A).

However, because we were unable to characterize the fragments resulting from aberrant splicing of the PHOX2B c.428G allele in terms of protein production, elongation (frame 2), or truncation (frame 3), we cannot provide a definitive conclusion regarding the consequences of this *PHOX2B* variant. Due to its location in the penultimate exon, we were unable to include exon 3 in the cassette cloned in the pSPL3 minigene, as it is not flanked at the 3′ end by an intronic sequence that would have allowed the evaluation of splicing between exon 2 and the pSPL3 acceptor site. Nonetheless, we cannot rule out partial no-stop-mediated mRNA decay in the case of “frame 2,” resulting in protein elongation and the subsequent degradation of the mutant mRNA allele.

This would be consistent with no known associations with NCC-derived tumors in patients carrying *PHOX2B* variants associated with the activation of mRNA surveillance (Zhou et al., 2021).

However, we cannot know *a priori* whether the mRNA of the mutant allele will be partially or completely degraded or, alternatively, fully produced in case the quality control is completely bypassed, nor in what type of translational context this might occur. We have summarized these hypotheses in a table showing the possible consequences of different classes of mutations of the PHOX2B protein (Table 3).

**TABLE 3:** *PHOX2B* variants described in terms of their possible consequences on the PHOX2B protein.

Analysis group	Variant category description	% Full-length protein	NMD?	NSD?
Missense and in-frame indels	• Missense variants that cause substitution of amino acids	100	No	No
	• Small in-frame indels	100	No	No
NMD variants	• Early loss-of function (LOF) variants that likely trigger NMD due to the presentation of PTCs in exon 1 or exon 2	<100	Yes	No
Non-NMD variants	• Variants in exon 3 that are not likely to trigger NMD	100	No	No
	• PTCs in exon 3, occurring before the second polyalanine tract, lead to a truncated PHOX2B protein with no polyalanine tract	100	No	No
	• Variants that lead to frameshift in exon 3 before the second polyalanine tract and disrupt the polyalanine tract, causing extension of the PHOX2B protein beyond the canonical stop	<100	No	Yes
	• Variants that lead to frameshift in exon 3 after the second polyalanine tract	<100	No	For frame 2
	• Variants that lead to truncation right after the second polyalanine tract	100	No	No
	• Variants that lead to loss of the full gene sequence	0	No	No
**Missense variants leading to frameshift**	**• Variants affecting exon 2–exon 3 splicing, thus leading to frameshift**	**≤100**	**No**	**For frame 2**
Loss of full exon/gene	• Synonymous change, does not lead to any protein coding change^a^	100	No	No

^a^ if in proximity of exon–intron boundaries, a splicing defect cannot be excluded.

NMD, nonsense-mediated decay; NSD, non-stop-mediated decay; “frame2” indicates elongated proteins.

the variant category reported here is emphasized in bold.

In any case, regardless of the modality and extent of this quality control, the defective splicing observed in association with the present PHOX2B mutation partially affects the homeodomain encoded between exons 2 and 3. This could have consequences in terms of the transactivation efficiency of target genes and, ultimately, could strengthen the hypothesis of a loss-of-function (LOF) mechanism attributed to this mutation (Figure 4B).

**FIGURE 5:**
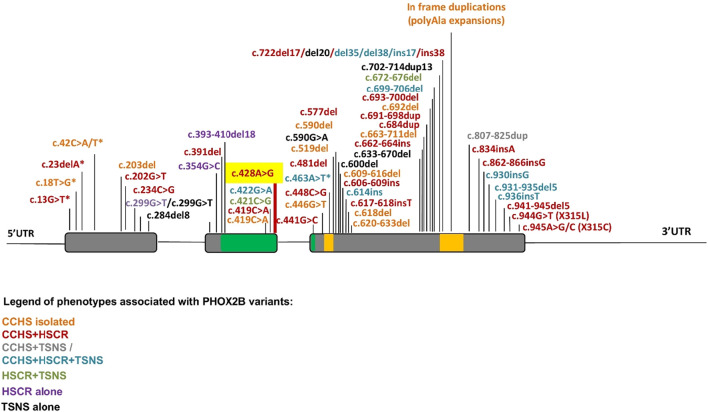
Summary of *PHOX2B* variants and associated phenotypes. The location of mutations along the *PHOX2B* gene identified in CCHS, HSCR, and neural crest-derived tumors (NCTs), and their possible combinations, are indicated by a color scale. Asterisks indicate a MS mutation leading to a premature stop codon. Gray: exons; green: homeodomain; yellow: 9 and 20 polyalanine domains.

Furthermore, different mechanisms underlying the co-occurrence or non-occurrence of neural crest tumors with CCHS and HSCR still require additional experimental confirmation. In contrast to the missense mutation in exon 2 not close to exon–intron boundaries, which are mostly associated with neural crest tumors, many FS mutations in the last exon 3 are associated with the CCHS + HSCR phenotype, probably because the aberrant hypomorphic protein can be produced when the degradation mediated by mRNA surveillance is partially or completely escaped (Figure 4B). Conversely, the incidence of neural crest-derived tumors increases with the presence of FS mutations toward the end of the gene (Figure 5) (Di Lascio et al., 2018), although the underlying mechanism remains unclear.

## 5 Conclusion

In conclusion, we propose that when a missense mutation in the homeodomain does not affect splicing, the PHOX2B mutant protein can be fully produced, thus being competent to exert its pathogenic, gain-of-function effects, which are probably ascribable to downstream aberrant transcriptional regulation and/or modified interactomes. On the other hand, when the splicing mechanism is compromised, another pathogenic mechanism, likely producing an aberrant hypomorphic protein, could account for the sole HSCR co-occurrence. Further studies are required to address these unresolved questions.

## Data Availability

The full data set presented in this article is not immediately available because much of the data does not address the purpose for which it was generated, namely, obtaining a molecular diagnosis at the PHOX2B locus. Requests for access to the data set should be directed to the corresponding author, Isabella Ceccherini.
